# Using Action Research and Peer Perspectives to Develop Technology That Facilitates Behavioral Change and Self-Management in COPD

**DOI:** 10.1155/2014/380919

**Published:** 2014-05-18

**Authors:** Catherine McCabe, John Dinsmore, Anne Marie Brady, Gabrielle Mckee, Sharon O'Donnell, David Prendergast

**Affiliations:** ^1^School of Nursing & Midwifery, Trinity College Dublin, 24 D'Olier Street, Dublin 2, Ireland; ^2^Intel Ireland Ltd., Collinstown Industrial Park, Leixlip, County Kildare, Ireland

## Abstract

*Background*. Behavioural change and self-management in patients with chronic illness may help to control symptoms, avoid rehospitalization, enhance quality of life, and decrease mortality and morbidity. *Objective*. Guided by action research principles and using mixed methods, the aim of this project was to develop peer based educational, motivational, and health-promoting peer based videos, using behavioural change principles, to support self-management in patients with COPD. *Methods*. Individuals (*n* = 32) living with COPD at home and involved in two community based COPD support groups were invited to participate in this project. Focus group/individual interviews and a demographic questionnaire were used to collect data. *Results*. Analysis revealed 6 categories relevant to behavioural change which included self-management, support, symptoms, knowledge, rehabilitation, and technology. Participants commented that content needed to be specific, and videos needed to be shorter, to be tailored to severity of condition, to demonstrate “normal” activities, to be positive, and to ensure that content is culturally relevant. *Conclusions*. This study demonstrated that detailed analysis of patient perspectives and needs for self-management is essential and should underpin the development of any framework, materials, and technology. The action research design principles provided an effective framework for eliciting the data and applying it to technology and testing its relevance to the user.

## 1. Introduction

Chronic obstructive pulmonary disease (COPD) is the 4th leading cause of death worldwide [1]. The pattern of care for patients with COPD often involves lengthy hospital admissions, followed by a return to the home environment with little follow-up support for the management of disease symptoms. This inevitably leads to the hospital readmission with acute symptom onset which is unsatisfactory for the individual and financially unsustainable for increasingly stretched health services.

Behavioral change and self-management in people with chronic illness may help to control symptoms, avoid unnecessary rehospitalization, enhance quality of life, and decrease overall mortality and morbidity [2]. Behavioral change techniques influence change by using motivating techniques, education, problem definition, collaborative goal setting/problem-solving, contract for change, continuing support, and evaluation [3].

Recent studies highlight the role that video and multimedia based information communication technologies (ICT) can play in COPD self-management. A comparative study revealed that individuals using a 19-minute video outlining the benefits of exercise followed by a 30-minute video exercise programme 4 days a week for 6 weeks had significantly improved physical and emotional functional capabilities [4]. This is important as the British Lung Foundation Survey revealed that less than 2% of COPD patients in the UK had access to a rehabilitation exercise programme [4].

A study on the use of video technology to provide motivation for exercise concluded that individuals with COPD showed improved quality of life (QoL), coping skills, and diminished fatigue when using a videotape series focused on motivation and exercise [5]. Two hundred fourteen COPD patients took part in this 3-tiered RCT (video intervention versus standard video versus control). Groups using the video intervention were twice as likely to change from sedentary states to moderate exercise compared to the control groups. The video intervention group saw an improvement in activities of daily living and was particularly effective at prompting exercise with 84% of patients reporting routine exercise at 9-month follow-up. This study shows a distinct correlation between improved health benefits, motivation, and behavioral change with video intervention, suggesting that videos provide an effective medium to disseminate knowledge and encourage self-management. A randomized controlled trial that measured the effects of an animated diagram and video-based online breathing programme for dyspnea in patients with stable COPD concluded that a video based online training programme resulted in improved pulmonary function, exercise capacity, and health status in the treatment group only [6].

The theory of innovation diffusion states that individuals are more likely to consider technology use when relevant to them [7]. Therefore it is important to design new technologies including content from a patient centric perspective with reference to the health condition and cultural context of the individual. Studies related to behavioral change and COPD show significant positive effects for self-efficacy and behavioral change on administration of a behavioral change programme [8]. This paper discusses findings from a project guided by action research principles that explored patient based self-management and technology based programmes for people living at home with COPD and concludes that action research principles can be an effective framework design for behavioral change video and multimedia content development.

The main aim of this project was to develop peer based educational, motivational, and health-promoting peer based videos, using behavioral change principles, to support self-management in patients with COPD.

An objective of the project was to explore the needs, sources of support, and experiences of people living at home in relation to their knowledge, ability, and readiness to self-manage their care needs in relation to COPD.

## 2. Method

Action research principles and a cross-sectional descriptive methodology were chosen as a suitable method of enquiry for the aim of this study. Action research is a process by which new knowledge is generated and used to achieve change [9]. The strength of this process of inquiry lies in its ability to generate solutions to practical problems. In this study the action research took place over 4 phases (Figure 3).

Ethical approval was provided by the relevant institutional ethics committee (Faculty of Health Sciences).

## 3. Action Research Process

**Phase 1**

The project began with a review of the literature to identify the main self-management needs and resources relevant to COPD in an ICT context. This was followed with completion of the demographic questionnaire and focus group/individual interviews to ascertain the knowledge, readiness for self-care, sources of support, and perceived care needs of people with COPD. Focus group and individual interviews were video recorded and content was used for development of self-management videos in Phase 2.

**Phase 2**

In conjunction with the data from the interviews and video recordings, existing educational resources currently accessed by the client group or available to meet their identified needs were reviewed. This data guided and informed the development of new educational, motivational, and health-promoting peer related videos specific to COPD. Eighteen videos representing the 6 key themes of knowledge, symptoms (prevention), self-management (practices), rehabilitation, support (including carers), and “Real Time Health” were produced. The theme of “Real time Health” was constructed to highlight the role of psychology and the constant need for individuals to manage their condition in a “real time” home environment.

**Phase 3**

Participants were invited to participate in follow-up focus group interviews to explore user views and satisfaction with the video material produced. They were shown a series of five, 3–5-minute videos representing areas of self-management based on data gathered in Phase 1. Participants were asked to discuss any perceived positive and negative aspects to each video as well as discuss any improvements they would like to be considered as part of the Phase 4 iteration process. The key issues that the participants commented on were that some content needed to be more specific and focused and videos needed to be shorter, to be tailored to level of severity of condition, to demonstrate “normal” day-to-day activities, to be positive, and to ensure that content is relevant to the Irish Health service.

**Phase 4**

Using feedback from Phase 3, a second and final stage of video development was conducted.

## 4. Participants

Treatment of COPD in Ireland is managed mainly through primary health care settings by general practitioners (GP) and community nurses. Admission to acute care facilities only occurs for exacerbations of symptoms and discharge is usually followed by attendance at a local rehabilitation programme which may last up to 6 weeks and GP visits as required. Until recently only informal local support groups provided information and social support to people with COPD; however, recently a national support organization has been established. For the purposes of this study, individuals living with COPD at home and involved in community based COPD support groups were invited to participate in this project. Study information sheets with contact details were distributed at community based COPD support group events. Eligibility criteria included a diagnosis of COPD, member of COPD support group, over 18, and English speaking. When eligible participants contacted the researcher specific sessions were organized for those who were interested in attending.

## 5. Instruments

Participants completed a brief questionnaire comprising 25 items which documented some sociodemographic characteristics of the participants. These included age, gender, medical insurance, medical/social history, comorbidities, BMI, perceived health status, independence rating, carer support, where COPD advice was obtained, confidence in self-management, and ICT use.

Focus group interviews were conducted at community locations convenient to the participants, with a researcher as a facilitator. There were 10 focus groups in total (5 in Phase 2 and 5 in Phase 3), with an average of 6 participants in each. The final total of focus group participants was 32 as participants in Phase 2 focus groups were the same as Phase 3. The themes explored in the interviews included experience, knowledge, self-management, social support, health professional support, social and personal life, quality of life, perceptions, cognitions, and confidence. Twelve individuals who participated in a focus group interview also completed an individual participant perspective interview, four of whom completed a second individual interview as part of the project's prolonged engagement methodology to gather in-depth information.

## 6. Data Analysis

**Figure 1:**
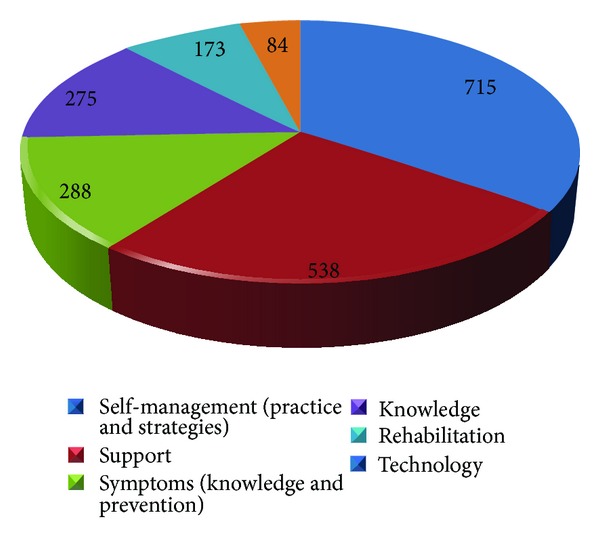
Core themes and reference totals extracted after coding analysis at Phase 1.

Descriptive statistics, using SPSS version 19, were used to describe the demographic data and included means, standard deviations, percentages, and ranges. Descriptive statistics were also used to describe the exploratory questions of the second part of the questionnaire. All recordings from the interviews were transcribed and transferred into NVivo version 9 for analysis. A constant comparative methodology facilitated the identification of patterns in participant responses and unifying concepts facilitating initial coding and development of core categories, which were revisited and consolidated, in line with the steps in the textural content analysis of [10]. Analysis continued until theoretical saturation was obtained and the number of references for each theme was calculated. Data from the individual interviews was analysed with the focus groups to triangulate findings at Phases 1 and 3, respectively (Figure 1). The content from these interviews was also used to construct the peer-perspective videos.

In order to ensure consistency in coded findings research team members used NVivo to manage and review transcribed data from the focus group interviews. Several COPD patients that gave important insights within an interview after coding were interviewed for a second time (prolonged engagement). This is in line with guidelines for ensuring trustworthiness in qualitative interviewing [11].

## 7. Results

Recruitment occurred from July to December 2011. Sociodemographic characteristics are outlined in Table 1. Thirty-two individuals participated in the initial focus groups at Phase 1 and again at Phase 2.

Coding of the transcripts from Phase 1 revealed 6 core categories relevant to COPD behavioral change. These included self-management, support, symptoms, knowledge, rehabilitation, and technology. Figure 1 outlines the number and portion of references attributed to each core theme across the focus groups and interviews.

Self-management was the main core theme discussed by participants followed by support. Management of symptoms and overall knowledge to the condition were almost equally referred to with rehabilitation methods the second last most talked about area. Each of these categories was further refined into various subthemes as outlined in Table 2.

### 7.1. Knowledge of the Condition

Participants distinguished between early stage knowledge immediately after diagnosis and knowledge obtained from years of living with the condition.

*FG: “In the beginning you would just have got it from a consultant or the doctor—probably looked up the net after that.”*

A distinct lack of online information related specifically to the Irish context was reported and also information was scattered across various sites rather than one site covering all areas of the condition effectively.

*I've been on the British Lung Foundation and different sites on the Internet but they do not give a proper breakdown of things. And most of it is American, which is different to us, they have different facilities to use and they call things a different name and you know it makes life a bit more difficult. You cannot do the same things as they do because we haven't got it, we might not understand the jargon you know, they talk a lot about medi-care which we haven't got. (DE)*

### 7.2. Symptoms

Participants reported 5 key subthemes in relation to the symptoms which were psychological symptoms, breathing difficulties, infections, symptom triggers, and relationship of symptoms with existing comorbidities. Psychological symptoms were broken down into 5 key branches: depression, isolation, frustration, embarrassment, and anger.

*FG: “You do feel isolated, when your first given, told the news I mean I just wouldn't accept it at all … and that just led to depression, just couldn't cope.”*

Patients who commented that they did not feel depressed also commented on “feeling fed up,” “worthless,” “thinking of euthanasia,” “disappointed,” “browned off,” “being vexed at why they got this,” “having a lack of motivation,” and little “joyfulness.”

Participants remarked that when breathing was good participants were optimistic, calm, and positive. If breathing was poor participants reported being conscious of the condition, struggling, panicking, gasping for air, and being frustrated.

*FG: “The shortness of breath in everything you do, just normal things like showering can be very frustrating.”*

#### 7.2.1. Self-Management

**Figure 2:**
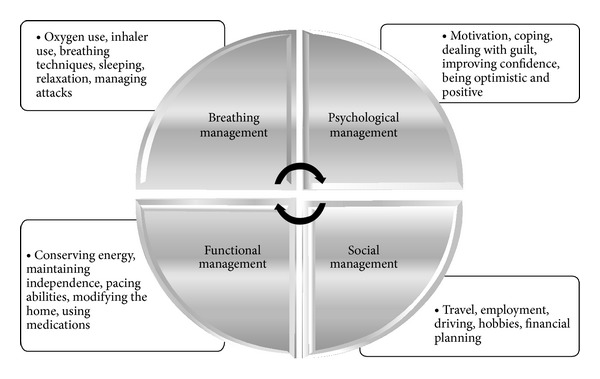
Four-way interactive structure for self-management.

**Figure 3:**
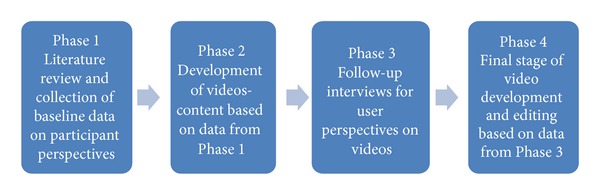
Action research process.

Self-management was the most referenced topic of conversation amongst both the focus groups and interviewees. Results produced a four-way interlogical model to patient's views of COPD self-management; if one area was positively affected, then all other areas replicated a positive outcome. The areas of breathing, functional, social, and psychological management (Figure 2) were seen as necessary to holistically self-manage in a primary care context.

*BS: “The first time I was told when I was doing the rehab program, that I needed to go on oxygen I was absolutely devastated because I thought how in the name of God am I going to live my life on oxygen. And I was really down in the dumps about it until I kind of got used to the idea. And then I said you know well if it helps, if it makes life easier, now it does not stop me getting out of breath but it helps me recover my breath quicker.”*

Functional management practices amongst participants in this study focused on the dispensation, conservation, and regulation of energy levels in order to maintain independence, which was referenced in relation to the ability to perform activities of daily living and social activities such as going to the local shop or cooking a meal.

*FG: “If I do decide to cut the grass myself and its only a small bit of grass I'd only do half of it and I'd have to leave it and come in and go out maybe in an hour later to finish it. I wouldn't have the energy to do it all in the one.”*

Tiredness and weakness associated with a lack of energy increased negative psychological responses to self-management.

*FG: “It affects your social end of it, because you just do not have the energy, you know if there's something going on and you are not feeling great well you just have to pass on it, you know.”*

The psychological processes behind COPD self-management revealed 5 core areas of importance for participants. These areas were patient motivation, coping ability/response, optimism or positivity, overcoming guilt, and confidence in self-management. Motivation was the most commented on area of importance referenced by participants followed closely by development of “coping” responses. Participants felt daily motivation to self-manage was difficult when they were feeling low or depressed, especially for those who were more socially isolated. Motivation was particularly difficult with regard to developing an exercise routine.

*Also very important and others have said it too, is to motivate yourself to do things. That can be difficult at times especially if you are feeling a bit sorry for yourself which you can be from time to time. (FG)*

Peer and family support appeared to be important in improving motivational drives to self-management.

* I set up a support group and from meeting those people at the support groups and I helped with a few support groups now and that gives me a sense of purpose or reason to do things. (ML)*

Learning to accept and live with the condition was referenced as key to participant's ability to cope; participants comment regularly that dwelling on their condition had an extremely negative impact on their motivation and confidence to self-manage.

* We all adjust to a different quality of life, even with age, its amazing … and my quality of life compared to what it used to be is what most people consider to be pretty awful but I do not, I'm happy with what I am able to do and I do it. (MA)*

The final subbranch to the psychological component of self-management was overcoming guilt. Participants almost exclusively felt guilt for their diagnosis and development of the condition. The foundation of this guilt was linked to regrets about previous behaviors such as smoking, which have been reported as a key to COPD onset. A consensus did emerge that their condition did not warrant sympathy due to the perception that the cause was self-inflicted.

*I felt guilt and feel slightly that my condition is my own fault. And I have a huge guilt trip about that. (FG)*

Confidence appeared linked to perceived independence as a key determinate of successful self-management.

*That's all part and parcel of your confidence so if you are independent you are going to have the confidence to go out and do exercises or whatever. (FG)*

Participants discussed 5 major areas of concern in relation to social activities; these include ability to drive, employment, hobbies, financial issues, and travel. Overall participant's felt that compared to pre-diagnosis their travel was now restricted and nonspontaneous particularly for those who required continuous oxygen therapy and needed to carry an oxygen tank.

*DE: “If I go away I've got to have the c-pap machine, the nebulizer and the portable oxygen concentrator. I mean that takes up a suitcase, you get all that lot together. So I cannot travel light anymore. That was my first gut reaction was oh no I'm going to be on this for the rest of my life now.”*

Additional problems associated with participant engagement in short-term travel included financial costs to fuel or public transport and the psychological feeling that they were restricting others' enjoyment.

Personal hobbies play an important role in maintaining confidence and independence as part of a social based self-management framework in COPD.

*Some nights I go and I might not get up and dance at all … if they are fast there's no way I could do them. But even if I do not do a dance I'm still keeping in touch with the friends that I made at the dancing over the years. (BE)*

Compensating for activities no longer possible is also central to participant coping strategies. Due to physical limitations several participants optimized current abilities and substituted new hobbies and practices.

*I just sit back and relax, no good getting annoyed about it, just take it, sit back and read the paper, turn on my wireless and listen to a bit of music, you know. (JO)*

The loss of employment was devastating for younger participants in this study and contributed to decreased confidence in self-managing. Participants commented on wishing to go back to work, while simultaneously acknowledging that the physical limitations and uncertainty of their illness progression would not allow them to do so.

* I would love to go back to work, I would love the purposefulness of it and the freedom of being able to earn your own money. I mean that's always been, I've always been to work up until I got the severity of this … after that I did not feel like I could go to work after that, kind of knocked my confidence a bit I suppose. (DE)*

All participants received fixed income benefits and/or a pension as their primary income and commented on the difficulty of living on a minimal budget that does not provide flexibility to support areas of change for the participant outside of daily living essentials.

*I'm fortunate my limited income just about covers what I have to do but it does not leave anything for holidays or anything out of the ordinary … I must admit I would like a holiday, that is one thing I really miss. (DE)*

Rehabilitation was referenced 173 times compared to self-management at 715. Four areas of rehabilitation, exercise, smoking cessation, medical rehabilitation (including pulmonary rehabilitation clinics), and diet, were referenced by participants in this study.

*I keep active. It can be tough at times but it has to be done. And the more you push yourself the better you feel anyway … I can see the difference; I'm that little bit more free. I can walk that little bit better. (ML)*

All participants consented that smoking contributed to the development of their condition and cessation of smoking was essential to rehabilitation and self-management strategies.

*I should have stopped, I did not, I kept smoking … even when I was on the inhalers, even on that I used to smoke. I smoked until I couldn't smoke. (JO)*

#### 7.2.2. Support

Support was the second most referenced area of concern for participants in this study (538 references) with health professional support, peer support, friends and family, and community/social support referenced in descending order.

*FG: “When you're in the system, in the hospital with the doctors and that and the nurses, they're brilliant.”*

*FG: “On the rehab course you get not just the proper exercises for your lungs but you also get a lot of education and a lot of information on the condition and also about the medication and all that sort of thing which you wouldn't pick up really.”*

*FG: “Once they understand it, once they accept you know that this is it and this is you for the rest of your life. I find my family is great now with me, I go everywhere with them.”*

#### 7.2.3. Technology

Technologies used by participants as part of their current self-management practices included the Internet (most commonly referenced), desktop/laptop computer, videos, and mobile phone. The Internet was labeled separately from computers due to its availability on multiple devices and platforms. Fluency and usage levels varied amongst participants with 50% of individuals having access to Internet based facilities. Internet usage focused on e-mail, resourcing knowledge, and using Skype, particularly for individuals isolated or with distant relations.

*DE: “Another thing about people on the internet you know at least if you look up what other people are going through you think oh I'm not that bad or you know it can make life a bit more bearable because during winter months I'm more or less indoors.”*

## 8. Discussion

International published trials on COPD self-management (including ICT use) suggest that self-management is linked to improved health status, reduced emergency visits to health professionals, and reduced hospitalizations [12–14]. A Cochrane systematic review of 14 controlled trials on self-management education in patients with COPD stipulated that it is not clear what influence self-management education has on COPD patients. While self-management reduces hospitalization, data is insufficient to formulate clear recommendations regarding what should comprise content in a self-management educational programme, to integrate skills into everyday patient life (e.g., diet, exercise, smoking cessation, and sleep habits) and how best this should be disseminated using new technology [13].

Developing a model to promote healthy lifestyle change after diagnosis of COPD is an extremely detailed task. Core to the development of this model is the integration of structures for increased engagement, improved patient motivation, and self-efficacy. This study demonstrates that detailed analysis of patient perspectives on their condition and needs for self-management is essential and should underpin the development of any framework, materials, and technology. Inclusion of users in the process of design, development, and usability of a technology based self-management programme ensures a more positive user experience. According to NICE guidelines the three key requirements for high quality care include effectiveness, safety, and user experience. Involvement of users in the design of a service or product opens up opportunities for design, function, and safety issues that may have been otherwise unapparent [15]. The action research design principles that guided this project provided an effective framework for eliciting user perspective data, applying it to technology in a patient centric manner and testing its relevance to the user. This type of approach may result in increased and sustained user compliance.

Knowledge of COPD at diagnosis appears critical to the outlook and coping responses invoked by patients. Time should be taken by health professionals to ensure patients are confident in their knowledge of COPD as soon as possible following diagnosis and commencement of treatment. Sites or dissemination material should be tailored to various severity groups in COPD; for example, those with mild/moderate disease appear not to engage with web sites showing the severe aspects of the condition. Peer support and learning should be ideally constructed within the cultural and health system in which a patient resides. Participants in this study expressed frustration that available online peer resources were developed outside of the Irish context, particularly in the United States, referencing services and situations unfamiliar to their self-management situation in Ireland.

Psychological related symptoms to living with COPD were commonly referenced areas in this study, supporting previous research suggesting that emotional status determines health status to a greater extent than lung function or exercise tests in COPD patients [16]. Participants in this study perceived social and peer support as central to alleviating negative psychological symptoms such as depression. Future research into improving the social support structure of individuals with COPD is recommended. In assessing psychological symptoms of COPD it is important to also review their level of social and peer support. Participant responses in this study suggest that depression amongst individuals in Ireland with COPD may be a “silent” or underreported phenomenon. Future research is recommended into this potential phenomenon as positive psychological perceptions are key to successful behavioral change and self-management.

Overall participants in this study believe that self-management is a unique and subjective process that one learns based on their personal experience of COPD. Key actions participants used in self-management focused around conserving energy, seeking support, avoiding triggers, and being motivated to change their lifestyle. Uncertainty amongst participants in relation to the progression of their condition means planning on a day-to-day basis only with key motivational drives to maintaining health orientated around family/social life and maintaining independence. Bartlett et al. [3] had similar findings in relation to the importance of planning daily goals for successful self-management. In dealing with “triggers” to symptom exacerbations participants revealed a willingness to modify behavior and sustain daily techniques to anticipate and deal with changes in their condition. Similar to Dinesen et al. this project recommends that designing a framework to allow individuals to share these techniques via peer learning online would be off benefit in potential behavioral change and self-management on a daily basis [17].

Participant responses in this study revealed a potential logical interrelated self-management model based on function, breathing, psychological, and social management, which requires further detailed research (Figure 2). In order to support and facilitate effective self-management and behavioral change in patients with COPD, all 4 areas of this model need to be addressed when developing programmes.

Combining educational learning materials on the use of assistive breathing technologies with correct relaxation and breathing techniques appears crucial to the foundation of an effective breathing management behavioral change programme. Exercise is essential to patient rehabilitation and behavioral change and when constructing an exercise based behavioral change programme two key elements need to be incorporated. Firstly, an extensive educational and illustrative component is required to facilitate effective learning of required exercises. Secondly, a motivational component needs to be included based on learning via social contact with peers, family, and friends to encourage and support behavioral change, for example, face to face contact, e-mails, or phone call. Programmes that provide both elements for patients with COPD are very effective but limited [17].

Participants who reported guilt feelings were less inclined to seek support when needed. This may hamper effective rehabilitation and self-management and is an issue that needs to be addressed as part of the patient rehabilitation process as patients who do not seek adequate support due to a “guilt complex” may have their recovery stifled and experience decreased quality of life. Methods to overcome feelings of guilt should be incorporated in all behavioral change or psychoeducational self-management programmes as these feelings may prevent patients seeking support. This continued subtheme of social support avoidance due to feelings of guilt needs to be examined and treated within the wider COPD patient population.

The symbolism of oxygen use with loss of independence is a major worry for individuals with COPD. Addressing this with patients at an early stage in diagnosis is important as part of a lifestyle change process that facilitates successful self-management behaviors. Practical advice on issues like travelling long and short distances with portable oxygen can help patients adapt more easily to living with COPD. Any behavioral change program to improve breathing apparatus use needs to address individuals issues of using apparatus in public, teach appropriate breathing techniques to supplement apparatus use and breathing regulation, and also teach relaxation techniques to effectively regulate breathing techniques and apparatus use especially in stressful or panic situations.

When developing and recommending self-management programmes, financial costs also need to be taken into consideration as most patients will be on fixed income health benefits and/or pension supports which may not be sufficient to finance lifestyle/home modifications. Self-management programmes should include information on all potential financial and health assistance subsidies available.

Augmenting self-efficacy is important in determining which tasks an individual will perform or avoid based on level of knowledge. This process of knowledge empowerment has been seen to assist in developing patient self-efficacy to make relevant decisions in self-management [18]. Self-efficacy plays a major role in explaining health related behaviors [19] and thus it is essential that it is factored into any outputs from a self-management or behavioral change programme. Available studies show significant positive effects for self-efficacy and behavioral change following a structured educational programme [8, 20] with improved self-efficacy noted to improve and increase engagement with particular tasks [21]. A recent pilot RCT has demonstrated that compliance with behavior change programmes using technology was found to be high which resulted in increased benefits in terms of self-management [22].

Commonly adopted behavioral change models used in COPD self-management based programmes include the Transtheoretical Model (TTM) which concentrates on processes of change and support required to adopt a healthier lifestyle [23]. Other social cognition and health belief models used in the study of health related behaviors in COPD include the Theory of Planned Behavior; the Theory of Reasoned Action; the Health Belief Model, the Attitude-Social Influence-Efficacy Model [24] and the Self-Efficacy Model [25]. These models focus primarily on patient self-efficacy, giving the patient confidence to carry out a behavior necessary to reach a desired goal. Key to achieving self-efficacy is the modeling of patient beliefs and perceptions around their condition and potential recovery. The use of action research as a framework in this project demonstrates its feasibility and effectiveness in ensuring patient beliefs/perceptions form the basis for a technological approach to facilitate self-management in COPD.

The authors of this study recommend further investigation into relationships between perceived psychological self-management of those with COPD and the functional based selection, optimization, and compensation theory of successful ageing. In-depth knowledge of these processes with the COPD population may be of benefit when tailoring a self-management program to this group.

A key strength of this study is its demonstration in the feasibility and importance of codevelopment/design of technology based programmes for people with COPD, in order to ensure that content is relevant, easy to use, and needs based. This will improve compliance and ultimately increase behavioural change and self-management.

## 9. Limitations

Recruitment of participants was solely from COPD support groups. Individuals in these groups were more proactive in researching and promoting COPD knowledge, lifestyle changes, and societal awareness.

## Figures and Tables

**Table 1:** Focus group participant profile.

Participants (32)			

Gender breakdown	47% male, 53% female		

	Mean	Standard deviation	Range

Average age	67	8.44	46–78
BMI	25.8	4.64	20.40–38.52

Medical coverage	Have complete medical cards (84%)		
	Covered by medical card for GP consultations (75%)		
	Covered for medications and prescriptions (47%)		
	Covered by private health insurance in their own name (34%)		
	Covered through a family member (6%)		

Past medical history breakdown (%)	TIA/stroke (3%)		
	Heart disease (16%)		
	Hypertension (38%)		
	Diabetes (6%)		
	High cholesterol (22%)		
	Anxiety and depression (31%)		
	Other (47%)		

Accommodation support (%)	Lived alone (38%); lived with nondependents (56%); lived with dependents (6%)		

Smoking cessation (%)	Did smoke but have quit (78%); still smoke (9%); nonresponders (13%)		

Perceived health status (%)	Fair (41%); average (41%); poor (13%); good (13%)		

Independence rating (%)	Required some assistance (63%); fully independent (31%); nonresponders (3%)		

Carer support (%)	Spouse (47%); children (9.4%); friend (3.1%); partner (3.1%); other (12.5%); remainder—nonrespondents		

Obtaining information (%)	Books/newspapers/magazines (21%); Internet (12.5%); Digital Recordings (6%); health professionals (69%); alternative sources (25%)		

Confidence in self-management (%)	Yes (72%); no (29%)		

ICT use (%)	Standard mobile phone (91%); smart phone (25%); desktop home computer (25%); laptop (25%)		

Internet connectivity (%)	Have access to Internet based resources (50%); have access to an Internet connection (53%); use Internet daily (25%); use Internet a few times a week (9%); use Internet a few times per month (6%)		

Confidence in ICT use (%)	Fully confident (50%); partially confident (16%); neutral (12.5%); not confident (6%); nonrespondents (16%)		

**Table 2:** Six core categories and subthemes.

Core theme	
Knowledge	Knowledge of the condition; knowledge of diagnosis; knowledge of the cause; awareness of COPD; stigma
Symptoms	Psychological; breathing related; infections; associated comorbidities and symptom triggers
Rehabilitation	Exercise; diet; smoking cessation; medical rehabilitation (including pulmonary)
Self-management (practice and strategies)	Breathing related; functional management; psychological; social management
Support	Health professional; peer support; friends and family; community and social support
Technology use	Internet; computers; videos; mobile phones; tablets
